# **Conventional radiography for the assessment of focal bone lesions of the appendicular skeleton:** **fundamental concepts in the modern imaging era**

**DOI:** 10.1007/s00256-024-04854-6

**Published:** 2024-12-24

**Authors:** George R. Matcuk, Leah E Waldman, Brandon K. K. Fields, Marco Colangeli, Marco Palmas, Alberto Righi, Giacomo Filonzi, Amandine Crombé, Paolo Spinnato

**Affiliations:** 1 Department of Imaging, S. Mark Taper Foundation Imaging Center, Cedars-Sinai Medical Center, 8700 Beverly Blvd, Ste M-335, Los Angeles, CA 90048 USA; 2 Department of Radiology, Duke University School of Medicine, Durham, NC 27705 USA; 3 Department of Radiology & Biomedical Imaging, University of California, San Francisco, San Francisco, CA 94143 USA; 4 Orthopaedic Oncology Unit, IRCCS Istituto Ortopedico Rizzoli, 40136 Bologna, Italy; 5 Department of Pathology, IRCCS Istituto Ortopedico Rizzoli, 40136 Bologna, Italy; 6 Department of Radiology, Ospedale Maggiore, 40133 Bologna, Italy; 7 Department of Musculoskeletal Radiology, Pellegrin Hospital, Bordeaux University, F-33000 Bordeaux, France; 8 Diagnostic and Interventional Radiology, IRCCS Istituto Ortopedico Rizzoli, 40136 Bologna, Italy

**Keywords:** Bone, Osseous, Appendicular skeleton, Tumor, Lesion, Benign, Malignant, Radiography

## Abstract

Bone lesions of the appendicular skeleton can be caused by primary benign or malignant tumors, metastases, osteomyelitis, or pseudotumors. Conventional radiography plays a crucial role in the initial assessment of osseous lesions and should not be underestimated even in this era of modern complex and advanced imaging technologies. Combined with patient age, clinical symptoms and biology, and lesion features including location, solitary versus multiplicity, density, margin (transitional zone evaluated with Lodwick-Madewell grading score), and, if present, the type of periosteal reaction and matrix mineralization can narrow the differential diagnosis or offer a likely diagnosis. These radiographic features help guide further follow-up or management.

## Introduction

Primary bone tumors are uncommon. Bone metastases far outnumber primary bone tumors. Benign bone tumors are around 100 times more frequent than malignant primary bone tumors [1]. Bone sarcomas account for only 0.2% of all malignancies [2]. The most common malignant tumors are osteosarcoma (1.68/million/year), chondrosarcoma (0.79/million/year), and Ewing sarcoma (0.76/million/year) [3].

Conventional radiographs remain key for the assessment of bone tumors and should still be ordered even when a lesion is initially identified on advanced imaging, such as computed tomography (CT), magnetic resonance imaging (MRI), or nuclear medicine studies. In combination with patient history and demographic information, radiographic evaluation of bone lesion location, shape, size, margins, periosteal reaction, and internal mineralization can lead to an accurate diagnosis (benign vs malignant) in over 80% of cases [4]. This review will detail a stepwise approach to the conventional radiographic assessment of bone lesions for each of these features and differential considerations.

## Bone neoplasms and focal bone lesions

### General considerations

Non-traumatic skeletal lesions can be classified into five main categories: benign tumors, intermediate (locally aggressive or rarely metastasizing) tumors, malignant tumors, osteomyelitis, and other non-neoplastic non-infectious (“pseudotumoral”) lesions.

Tumors arise from autonomous, atypical, and progressive growth of cells within bone tissue. Tumor classification is histological and based on the type of cells composing the tumor and the tissue they produce [5]. For instance, if a tumor consists of osteoblasts/osteocytes and produces osteoid matrix, it is classified as osteosarcoma if malignant and osteoid osteoma or osteoblastoma if benign (depending on its size). Bone lesions are divided into:

- **Benign bone tumors** exhibit autonomous growth but, in general, at a slower rate compared to malignant tumors. Cellular morphology remains relatively typical and recognizable rather than disorganized as in malignant tumors. Tumor cells tend to differentiate and partly retain their specific function. Their growth is expansive and delimited by a proper capsule. Depending on their biological aggressiveness, they are categorized as latent/inactive, active, or aggressive [6]. Benign tumors do not metastasize and rarely recur locally. These tumors are generally managed based on their size, location, and symptoms. Examples of benign bone tumors include: osteoid osteoma, enchondroma, osteochondroma, fibrous dysplasia, non-ossifying fibroma, hemangioma, simple bone cyst and aneurysmal bone cyst [6].
- **Intermediate (locally aggressive or rarely metastasizing) tumors **exhibit slow but more progressive growth compared to benign tumors. They have an invasive growth pattern rather than expansive. Therefore, if not completely removed, including a layer of surrounding healthy tissue in some specific cases, they tend to recur locally while infrequently metastasizing [7]. Examples of intermediate tumors include: atypical cartilaginous tumor, osteoblastoma, desmoplastic fibroma, epithelioid hemangioma, osteofibrous dysplasia-like adamantinoma, mesenchymoma, and giant cell tumor of bone (GCTB) [7]. In fact, GCTB, once considered among benign tumors, has been classified by the World Health Organization since 2020 as an intermediate tumor, neither completely benign nor definitively malignant due to frequent local recurrences and rare pulmonary metastases [5].
- **Malignant bone tumors** generally exhibit rapid growth over time. Cellular morphology is often atypical, and tissue architecture is often disrupted. They have an invasive growth, patterns, often not delimited by a proper capsule but by a surrounding reactive tissue pseudocapsule permeated by satellite tumor nodules. Morphologically, malignant tumors show significant cytological atypia often associated with high mitotic activity and presence of tumoral necrosis. They can recur locally and metastasize distantly [8]. In contrast to malignant soft tissue tumors, where the French Federation Nationale des Centres de Lutte Contre le Cancer (FNCLCC) is used and has been validated, in malignant bone tumors, the histological subtype often determines the grade. For example, Ewing sarcoma, mesenchymal chondrosarcoma, angiosarcoma, dedifferentiated chondrosarcoma and the majority of osteosarcoma variants (conventional, telangiectatic, small cell, secondary, high-grade surface) are always considered high grade, whereas clear cell chondrosarcoma, parosteal osteosarcoma, low-grade central osteosarcoma and epithelioid hemangioendothelioma of bone are classified as low-grade [5].The exceptions are conventional chondrosarcoma, that is often graded using the 3-grading system proposed by Evans et al. [9], and adamantinoma and conventional chordoma that are not graded for definition and are only considered malignant neoplasms.
- **Osteomyelitis** is an inflammatory process of the bone sustained by pyogenic organisms, most commonly bacteria. Excluding post-traumatic and post-surgical forms, infections are distinguished into 3 main groups: a) hematogenous forms in which bacteria reach the bone via the bloodstream originating from infectious foci mainly located in the rhino-pharyngeal mucosa, upper respiratory tract, or urinary tract; b) primary chronic osteomyelitis such as Brodie's abscess; c) tubercular osteoarthritis [10].
- **Non-neoplastic non-infectious bone lesions** include all those entities that radiologically resemble a tumor even though they are not. Most are incidental findings during examinations for other conditions but in some cases, they can be clinically symptomatic too. Examples of pseudotumoral bone lesions are subchondral cysts/geodes, giant cell reparative granuloma, and brown tumors of hyperparathyroidism [11].

### Epidemiology

There is limited data available regarding the epidemiology of benign bone lesions, but it is generally accepted that such lesions are significantly more frequent than malignant bone tumors of the skeleton. Benign bone tumors are often diagnosed in young individuals, with a peak incidence in the first two decades of life. It is rare for a benign tumor to be diagnosed for the first time after the age of 40. The male/female ratio for benign tumors is approximately 1.5:1 [5–8].

The most frequently encountered benign bone tumors include osteochondromas, osteoid osteoma, aneurysmal bone cyst (ABC), chondromas, fibrous dysplasia, and chondroblastoma. Non-ossifying fibroma (NOF) and simple bone cysts, previously considered pseudotumoral lesions, are now classified under specific tumor categories. For instance, NOF and ABC are classified as osteoclastic giant cell-rich tumors. Although benign tumors can theoretically arise throughout the skeleton, some histotypes have characteristic localizations. For example, simple bone cysts predominantly localize in the metaphyseal regions of long bones, enchondromas in the tubular bones of the hands, and chondroblastomas in the epiphyses of long bones. Giant cell tumor of bone, currently considered an intermediate-grade neoplastic lesion, tends to localize more frequently in the meta-epiphyseal eccentric region of long bones such as the distal femur, proximal tibia, and distal radius [5–8].

Regarding malignant skeletal lesions, the most frequent ones are represented by carcinoma metastases and lesions associated with hematological diseases such as multiple myeloma and lymphoma, predominantly in adulthood and the elderly [1]. Primary malignant skeletal bone tumors, commonly called sarcomas, are very rare and globally represent about 0.2% of all neoplasms with an incidence of 0.8–1 cases per 100,000 inhabitants per year [1]. Osteosarcoma is the most frequent histotype among primary malignant bone tumors with an incidence of 0.3 new cases per 100,000 inhabitants per year [1]. Chondrosarcoma is the second most frequent histotype with a similar incidence to osteosarcoma, followed by Ewing’s sarcoma with an incidence of 0.1 new cases per 100,000 inhabitants per year [1]. Unlike metastatic lesions, sarcomas are more frequently diagnosed in pediatric patients and young adults. Specifically, Ewing’s sarcoma is more common in children under 15 years old and osteosarcoma in adolescents between 15 and 19 years [1]. On the other hand, chondrosarcoma has a higher incidence between 30 and 60 years old [8].

## The role of conventional radiography in the initial assessment of focal bone neoplasms

### American College of Radiology recommendations

According to the ACR, conventional radiography is usually appropriate as the initial imaging study when a bone neoplasm is suspected [12]. If the lesion is radiographically occult, MRI without or with and without contrast should be the next step in symptomatic patients [12]. CT is typically utilized in the evaluation of suspected osteoid osteoma and may be appropriate to evaluate lesions that have an indeterminant or aggressive radiographic appearance to better delineate endosteal scalloping or cortical destruction/breakthrough or pathological fracture and to better characterize matrix mineralization patterns [12]. CT is also useful for deep locations (pelvic bone, spine, skull base) or when there is a contraindication for MRI. Conventional radiography also plays a role in evaluating a lesion found incidentally on CT or MRI that is not definitely benign [12].

### Limits and advantages of conventional radiography

The advantages of conventional radiography in the initial assessment of focal bone neoplasms lie in its ability to estimate the rate of lesion growth in a single set of images acquired at one point in time, and by proxy, biological aggressiveness [13]. Other advantages of radiography in assessing bone lesions include wide availability and low cost [14].

Radiography is limited by relative lack of soft tissue contrast and inability to predict tumor extent in intact bone, making it an inappropriate choice for staging [12, 14]. Certain anatomic areas are poorly visualized due to overlapping structures which can also limit the use of radiography. Compared to CT, radiography has a lower sensitivity for identifying mineralized internal matrix or nondisplaced fractures [14].

### Lesion location

Just as British real estate tycoon Harold Samuel once coined, one of the keys to an appropriate differential diagnosis for bone lesion is “location, location, location.” The specific bone of involvement, type of bone (flat versus long), and longitudinal and transverse location within a long bone all help to narrow down the list of likely entities.

#### Part of the appendicular skeleton

**Fig. 1:**
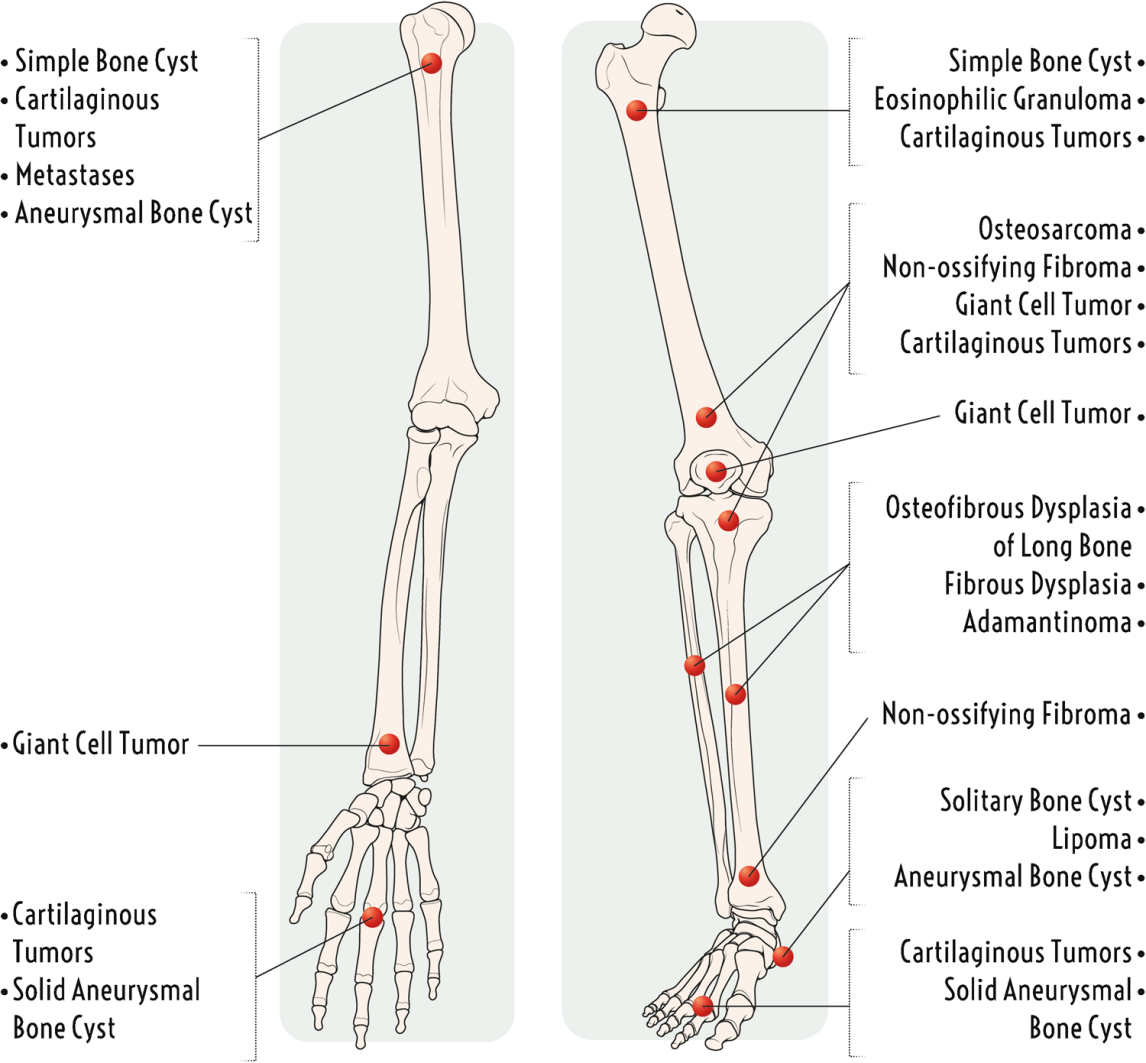
Illustration demonstrating predilection of some tumors for specific bones in the appendicular skeleton

Some bone tumors have a predilection for specific bones in the appendicular skeleton (Fig. 1) [15]. A classic example is adamantinoma, which involves the tibia in 80–85% of cases [16]. Some lesion locations have a limited differential diagnosis. For example, a well-defined lucent lesion of the calcaneus has a classic differential diagnosis of unicameral bone cyst, intraosseous lipoma, intraosseous ganglion, and pseudotumor (also known as pseudocyst due to decreased trabeculae in Ward’s triangle) [17]. A central versus a peripheral location in the appendicular skeleton can be helpful for assessing the risk of malignancy in chondroid lesions, with chondrosarcoma common and enchondroma unusual in the pelvis and vice versa in the hands or feet, although there is an overlapping frequency in the long bones [18]. Most appendicular bone tumors are found around the humerus and knee (distal femur or proximal tibia) [19].

#### Flat versus long bones

Much of the difference in predilection for flat versus long bones is based on the distribution of hematopoietically active red marrow, which is primarily located in the axial skeleton, pelvis, and proximal humeral and femoral metaphyses in an adult after conversion to fatty yellow marrow in the long bone epiphyses followed by diaphysis, and finally distal and then proximal metaphyses, typically by 25 years of age [20, 21]. Many tumors have a predilection for red marrow. For example, Ewing sarcoma typically involves the diaphyses of long bones in skeletally immature patients but shifts to flat bones in patients over 20 years old [15, 22]. Metastatic disease, lymphoma, and myeloproliferative disorders such as multiple myeloma and leukemia also typically involve red marrow [21]. Some primary bone tumors, such as osteosarcoma, prefer areas of rapid bone growth and therefore tend to originate near the physes of long bones [23].

#### Longitudinal location (epiphyseal, metaphyseal, or diaphyseal)

**Fig. 2:**
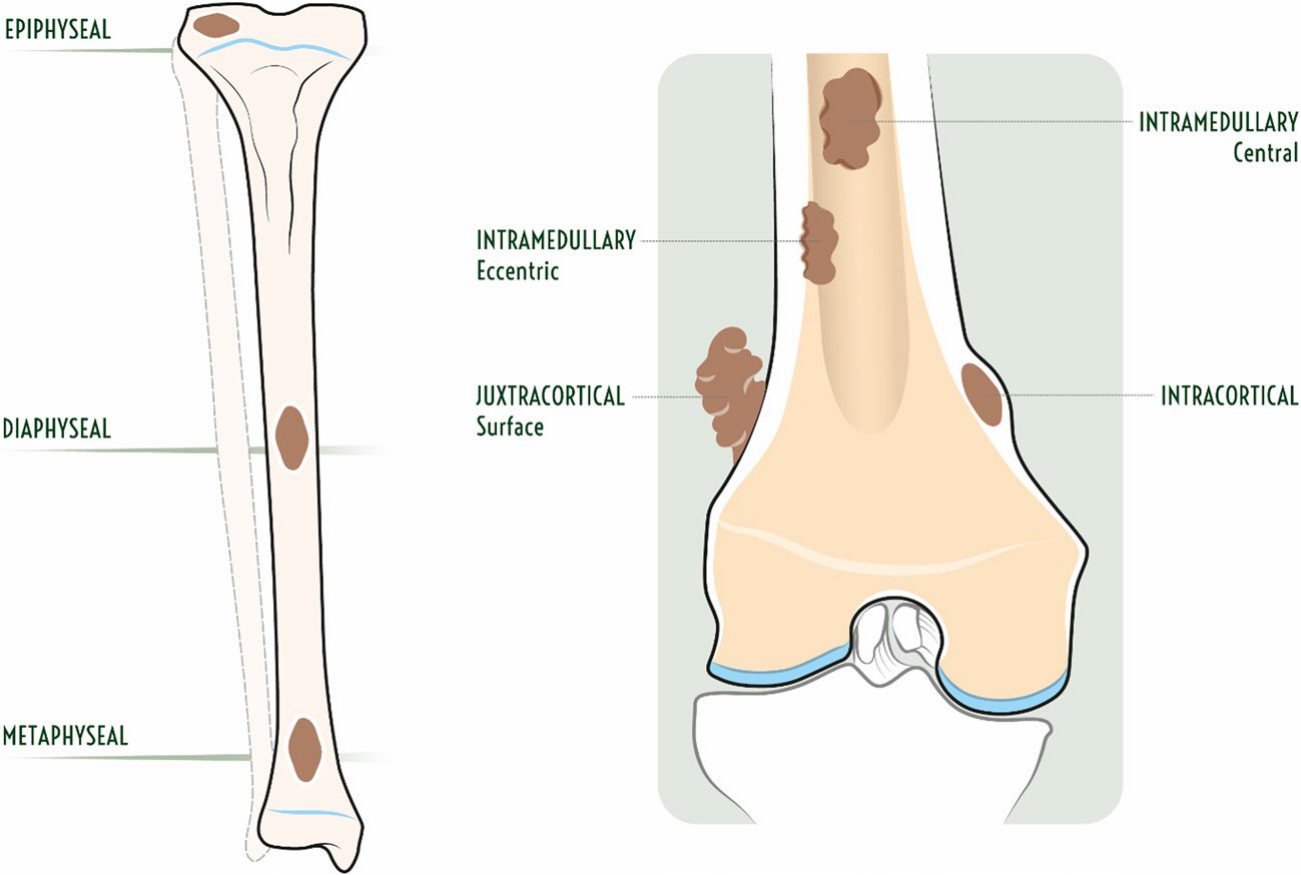
Illustration demonstrating potential longitudinal (epiphyseal, metaphyseal, or diaphyseal) and transverse locations [intramedullary central vs eccentric (endosteal), cortical, or juxtacortical (surface)] of bone tumors within a long bone

**Fig. 3:**
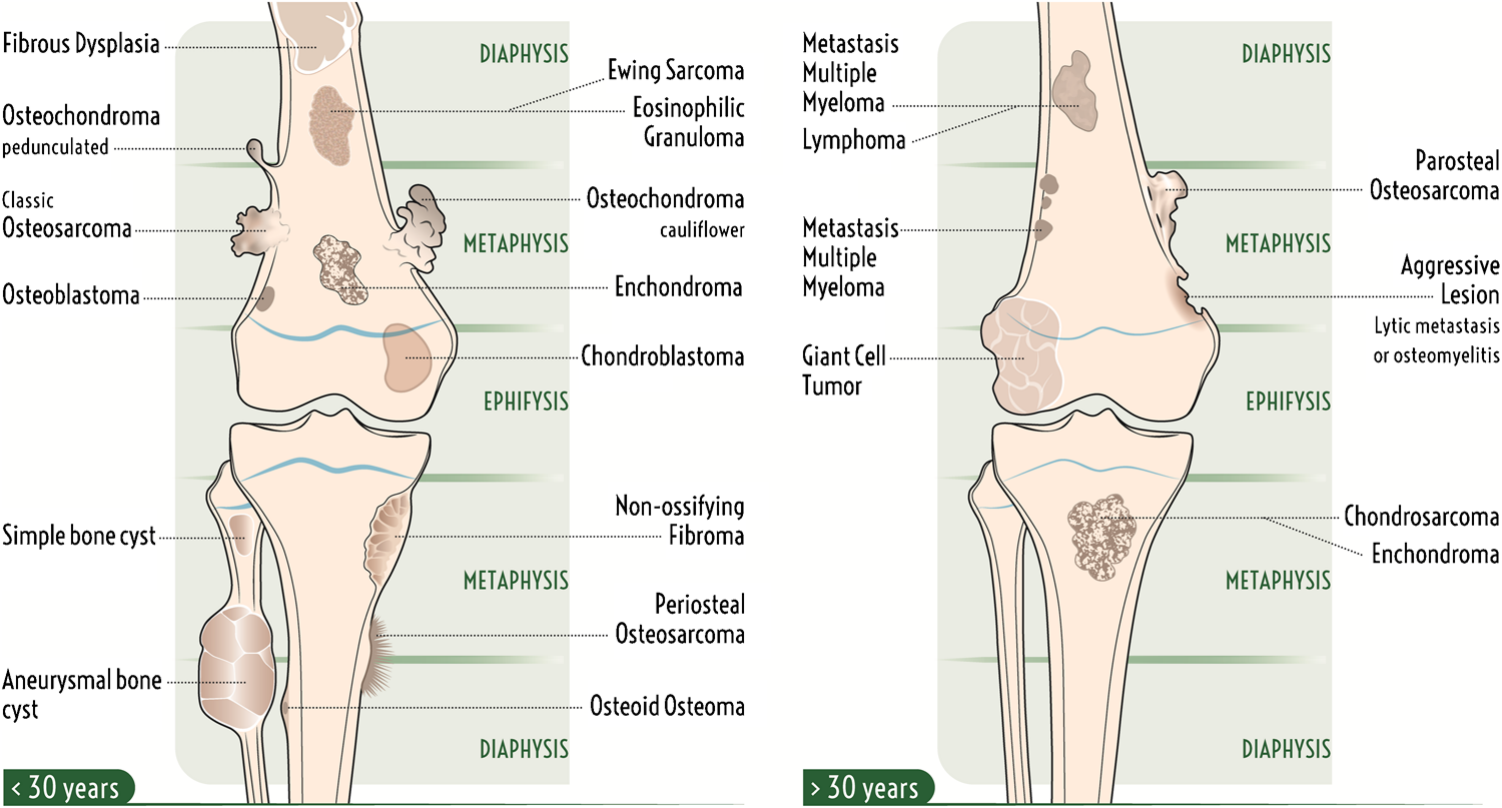
Illustration demonstrating classic longitudinal and transverse locations of different bone lesions in long bones typical for patients under 30 years old versus over 30 years old

Bone lesions that involve long bones tend to have a characteristic longitudinal location within the epiphysis, metaphysis, or diaphysis (Figs. 2 and 3). Most bone tumors occur in the metaphysis, both due to residual red marrow and rapid cell turnover in this region [15]. Osteosarcoma is the classic example of a metaphyseal lesion arising from osteoblasts that produce osteoid at the margin of the physis and metaphysis [15]. Osteochondromas and chondrosarcoma are also lesions that are typically metaphyseal, but as most bone lesions are metaphyseal (including many that can also occur in the epiphysis or diaphysis), it is easier to remember the lesions that can commonly be epiphyseal or diaphyseal [15].

Epiphyseal lesions include chondroblastoma (typically prior to growth plate closure), giant cell tumor (GCT; typically originating in metaphysis but extending into subchondral bone after growth plate closure), geode (intraosseous ganglion or subchondral cyst), and infection (Brodie abscess) [24, 25]. In patients over 40 years old, clear cell chondrosarcoma and Paget’s disease should also be considered in the differential diagnosis [26]. Apophyses, such the greater trochanter and tibial tuberosity, are considered epiphyseal equivalents, with the same tumor tendencies [26].

Bone lesions with a predilection for the diaphyses of long bones include unicameral bone cyst (migrated from metaphysis), adamantinoma, osteofibrous dysplasia, eosinophilic granuloma, Ewing sarcoma, fibrous dysplasia, and osteoid osteoma [15, 26]. Chondromyxoid fibroma and non-ossifying fibroma tend to occur near the metadiaphyseal junction [19]. In patients more than 40 years old, metastases, lymphoma, and multiple myeloma should be included in the differential diagnosis for both metaphyseal and diaphyseal lesions [19].

#### Transverse location (central, eccentric, intracortical, or juxtacortical)

Bone lesion can also be subdivided based on the transverse location with a bone as central intramedullary, eccentric intramedullary, intracortical, or juxtacortical (Figs. 2 and 3). Most bone lesions start as central intramedullary lesions, including solitary and aneurysmal bone cysts, EG, enchondroma, fibrous dysplasia, metastases, lymphoma, and multiple myeloma [19]. Eccentric lesions include osteosarcoma, chondroblastoma, chondromyxoid fibroma, and GCT [19]. Cortical lesions include fibroxanthoma, osteofibrous dysplasia, adamantinoma, and osteoid osteoma [23]. Juxtacortical (surface) lesions include osteoma, osteochondroma (with cortical and medullary continuity), periosteal chondroma, parosteal lipoma, bizarre parosteal osteochondromatous proliferation (BPOP or Nora’s lesion), and parosteal, periosteal, and high-grade surface osteosarcoma [27, 28].

### Solitary versus multiple lesions

**Fig. 4:**
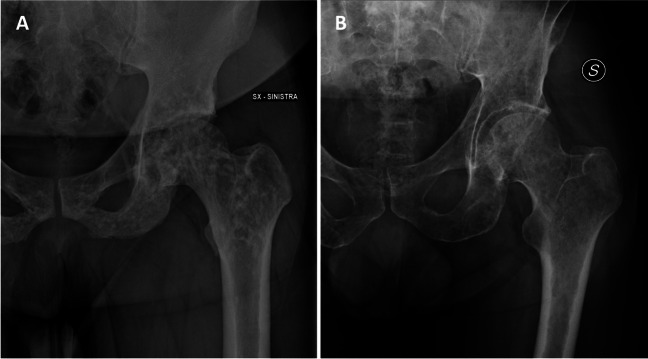
Radiographic examples of multiple sclerotic lesions (**a** osteopoikilosis) and multiple lytic lesions (**b** multiple myeloma) of the pelvis

Most primary bone lesions will be solitary; however, patients presenting with polyostotic (multifocal) bone lesions have a more limited differential diagnosis (Fig. 4).

Benign conditions that can present with multifocal lytic osseous lesions include brown tumors of hyperparathyroidism, amyloidosis, and chronic recurrent multifocal osteomyelitis (CRMO; also known as chronic non-bacterial osteomyelitis or CNO) [29]. Polyostotic fibrous dysplasia (including McCune-Albright syndrome with café au lait spots and endocrine disorder and Mazabraud syndrome with soft tissue myxomas), Hereditary Multiple Exostoses (HME or osteochondromatosis), Ollier disease (enchondromatosis) or Maffucci syndrome (multiple enchondromas and soft tissue hemangiomas), and Paget disease of bone are all disorders with multifocal benign osseous lesions with increased malignant potential [29]. Langerhans Cell Histiocytosis (LCH), formerly separated into eosinophilic granuloma (single or few), Hand-Schüller-Christian disease (multifocal bone lesions and lymph node and skin involvement), and Letterer-Siwe disease (disseminated multisystem involvement), is a clonal proliferation of histiocytes that is classified as intermediate (locally aggressive) with multifocal osseous involvement in 20% of cases [30].

Metastatic disease to bone is the third most common site after the lungs and liver and is polyostotic in over 90% of cases [31]. Multiple myeloma is the most common malignant primary bone tumor with multifocal osseous involvement in greater than 90% of cases (solitary lesions are referred to as plasmacytoma), although 30 − 50% of the bone must be destroyed before lytic lesions become apparent on radiographs [32, 33]. Leukemia and lymphoma can also have multifocal osseous lesions, although these lesions can be radiographically occult [20]. Osteosarcoma, Ewing sarcoma, and osteomyelitis can also be multifocal, particularly in children [34].

### Density

**Fig. 5:**
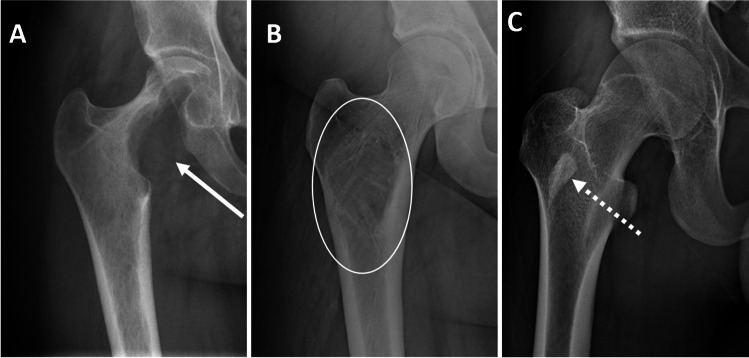
Radiographic examples of lucent (**a** arrow — giant cell tumor), mixed (**b** circled area — fibrous dysplasia), and sclerotic (**c** dotted arrow — bone island) of the proximal femur

The radiographic density (lucency or sclerosis) of a lesion is largely determined by relative differences in stimulation of osteoclasts (osteolytic) and osteoblasts (osteoblastic) by the tumor (Fig. 5) [23].

#### Lucent/lytic

Bubbly (well-defined) lucent lesions of bone have a differential diagnosis that can be remembered using the classic mnemonic (cover more than 95% of such lesions), FEGNOMASHIC (which can also be reordered to FOG MACHINES): Fibrous dysplasia, Enchondroma and eosinophilic granuloma, Giant cell tumor and geode, Non-ossifying fibroma, Osteoblastoma, Metastases and myeloma, Aneurysmal bone cyst, Simple bone cyst, Hyperparathyroidism (brown tumor) and hemangioma, Infection (osteomyelitis), and Chondroblastoma and chondromyxoid fibroma [35]. Lytic lesions that can be ill-defined also include Langerhans cell histiocytosis, giant cell tumor, osteomyelitis, metastases, multiple myeloma, Ewing sarcoma, osteosarcoma, chondrosarcoma, leukemia, and lymphoma [19].

#### Mixed

Bone lesions with a mixed lucent (lytic) and sclerotic (blastic) appearance have a more limited differential diagnosis. These include Paget disease of bone, fibrous dysplasia, osteofibrous dysplasia, adamantinoma, early bone infarct, and some metastases (particularly breast, lung, and prostate carcinoma) [19]. The mixed (middle) phase of Paget disease occurs after the lytic phase with decreased osteoclastic and increased osteoblastic activity, resulting in the four cardinal features of advancing osteolysis, trabecular coarsening and thickening, cortical thickening, and osseous expansion [36, 37]. In long bones, this manifests as advancing osteolysis of the diaphysis and sclerosis of the epiphysis and metaphysis, and in the pelvis as sclerosis of the iliopectineal and ischiopubic lines with enlargement of the pubic rami and ischium [38]. Fibrous dysplasia classically has varying degrees of hazy density (ground glass appearance) on radiographs [39]. Osteofibrous dysplasia, osteofibrous-like adamantinoma, and classic adamantinoma are a spectrum of fibro-osseous lesions of bone ranging from benign to malignant typically located in the mid tibia as expansile cortically based lesions with variable degrees of osteolysis and osteosclerosis [40]. Breast cancer is the most common metastasis to present with a mixed lytic/blastic appearance, occurring in 40% of cases [41]. Breast cancer metastases treated with chemotherapy are more likely to have mixed or sclerotic appearances and are more likely to be asymptomatic (66%) [42].

#### Sclerotic/blastic

Sclerotic bone lesions include bone island (enostosis), stress/insufficiency fracture, bone infarct (late), chronic osteomyelitis, osteoma, osteoid osteoma, healed non-ossifying fibroma, Paget disease (late phase), and osteoblastic metastases, particularly prostate, breast, lung, gastrointestinal, carcinoid, and transitional cell carcinoma [19, 43]. Multifocal and diffuse osteosclerosis should be considered a separate category that includes vascular (bone infarcts), neoplastic (osteoblastic metastases), metabolic (Paget disease and hyperparathyroidism/renal osteodystrophy), myeloproliferative (mastocytosis and myelofibrosis), drug-related (hypervitaminosis D and fluorosis) and developmental/other (osteopoikilosis, pyknodysostosis, sickle cell disease, sarcoidosis, tuberous sclerosis, and melorheostosis) causes [43–45]. The differential diagnosis for sclerotic solitary (focal) osseous lesions is primarily based on patient age, lesion location (intramedullary, cortical, or juxtacortical), degree of homogeneity, and aggressiveness, (tumorous versus non-tumorous), as discussed in other sections [46]. CT attenuation thresholds have been proposed with a mean attenuation of 885 HU and maximum attenuation of 1060 HU to differentiate enostoses from untreated osteoblastic metastasis, with sensitivities of 95% and specificities of 96%, although this decreases with limited accuracy of 61% when applied to all benign sclerotic bone lesions [47, 48].

### Transition zone (margin) assessment

The margin of a lytic bone lesion can be a marker for the aggressiveness and predictive of tumor growth rate. A grading system was first proposed and later modified by Lodwick and subsequently simplified and formalized by Madewell and is commonly used in practice [13, 49, 50].

**Lodwick-Madewell grading and revised systems**

**Fig. 6:**
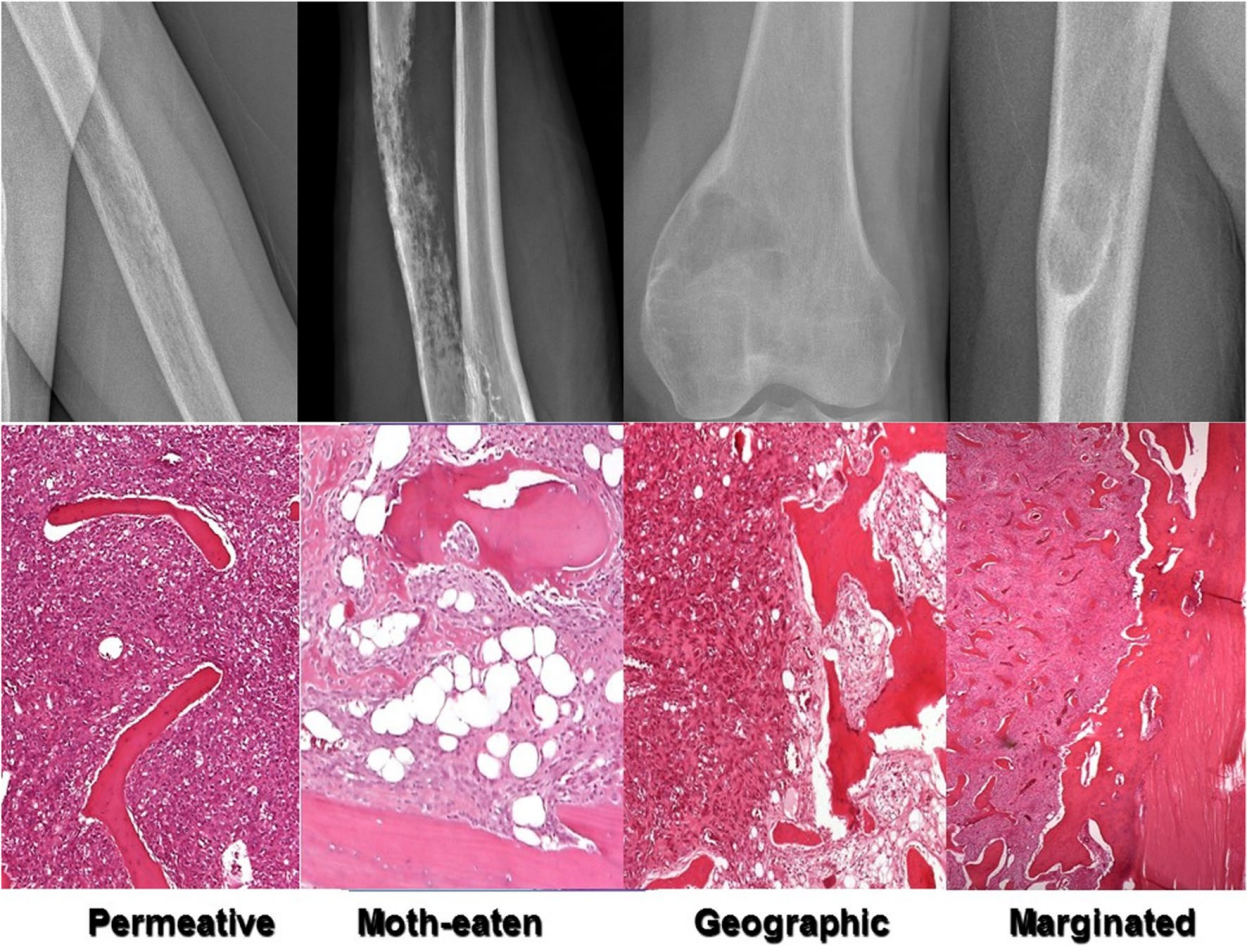
Radiographs (top) and histologic images (bottom) demonstrate different patterns of bone alteration and margins according to the Lodwick-Madewell grading system

•Type I — geographic
- These include non-aggressive bone tumors, which are round or ovoid in shape (geographic), and are divided into three sub-categories (Fig. 6) [50]:
•Type IA — well-defined sclerotic border
- Type IA lesions are the least aggressive, have a narrow zone of transition, and well-defined sclerotic margin [50]. A classic example is fibroxanthoma (also known as fibrous cortical defect if < 2 cm in size or non-ossifying fibroma if > 2 cm in size) [14, 51]. Other lesions that can have this appearance include Brodie abscess, unicameral bone cyst, fibrous dysplasia, intraosseous lipoma, chondroblastoma, chondromyxoid fibroma, and osteoblastoma [19].
•Type IB — well-defined without sclerotic rim
- Type IB lesions also have a narrow zone of transition, but lack a sclerotic rim [50]. These margins indicate indeterminate biologic potential and although usually seen with benign lesions such as giant cell tumor, enchondroma, and aneurysmal bone cyst; however, malignant lesions such as low-grade chondrosarcoma, metastases, and multiple myeloma can also have a similar appearance [14, 19].
•Type IC — ill-defined wide zone of transition
- Tumors with Type IC maintain a geographic (round or ovoid shape) but the margins are more ill-defined and indistinct with a wide zone of transition, indicating a more aggressive tumor [50]. Although benign lesions such as aggressive giant cell tumors may have this appearance, most Type IC lesions are malignant, such as chondrosarcoma, osteosarcoma, metastases, and multiple myeloma [19, 52].
•Type II — moth-eaten
- Type II lesions are non-geographic with ill-defined fields of bone destruction and a “moth-eaten” appearance with numerous foci of osteolysis that may vary in size and shape, though with a relatively intact cortex [14]. Although osteomyelitis may have this appearance, most of these aggressive lesions are malignant, including Ewing sarcoma, fibrosarcoma, angiosarcoma, osteosarcoma, lymphoma, and metastases [19].
•Type III — permeative

Type III lesions are the most aggressive with non-geographic permeative osteolysis, a wide zone of transition, and fine or fuzzy appearance, often including cortical destructive change. The differential diagnosis includes the same entities that can have a Type II appearance [14, 19].

**Fig. 7:**
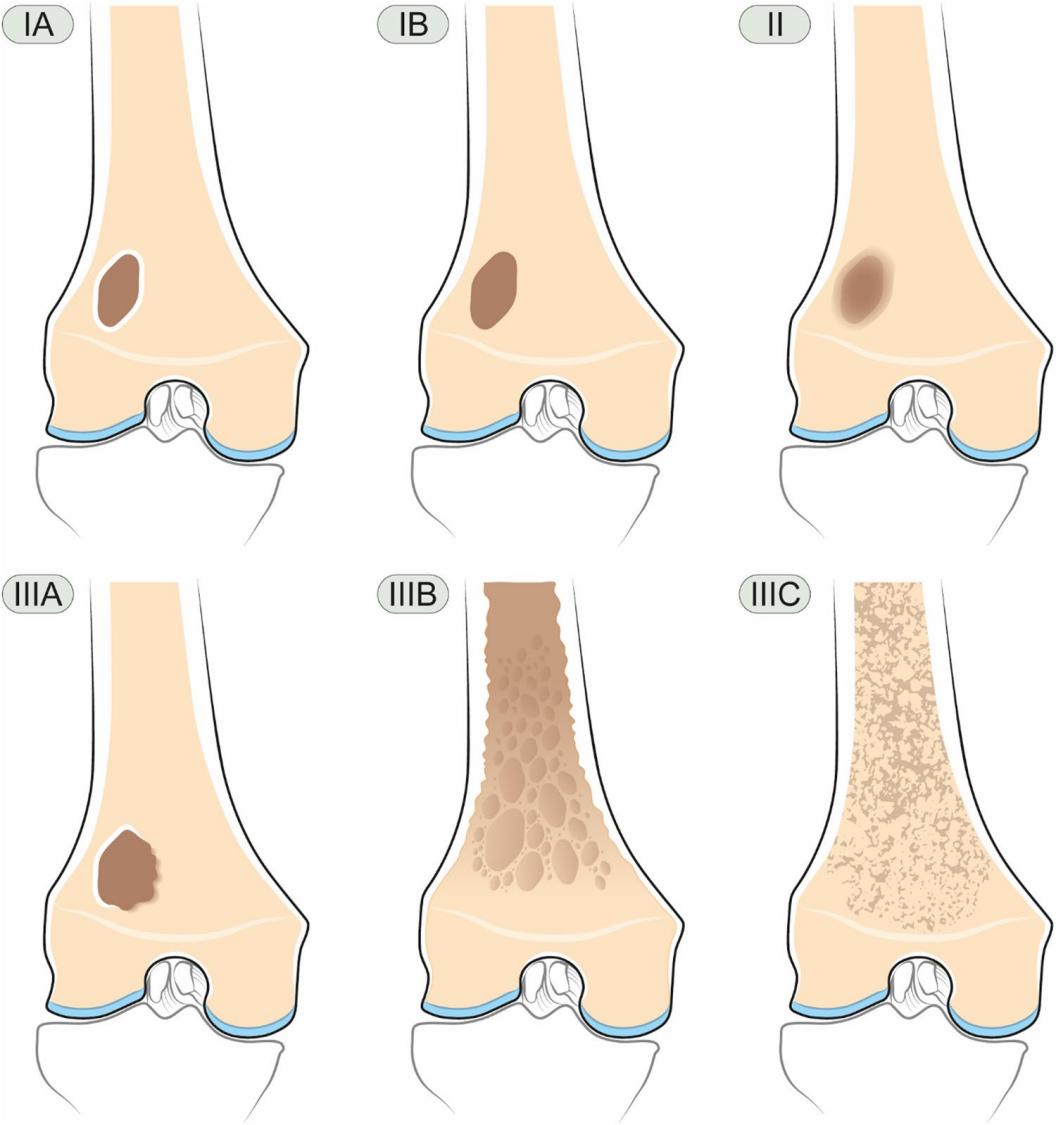
Illustration demonstrates the proposed modification of the Lodwick-Madewell grading system by Caracciolo et al.: type IA: geographic with sclerotic border; type IB: geographic without sclerotic border; type II: geographic but ill-defined margins; type IIIA: changing margin on serial radiographs; type IIIB: moth-eaten; and type IIIC: permeative

Caracciolo et al. proposed a modified system that combines types IC and II and redefines type II as geographic lytic lesions with partial or circumferential ill-defined margins and provides new subclassifications for type III as A: focal change in margin, changing margination, or progressive endosteal scalloping on serial radiographs; B: moth-eaten and permeative patterns of osteolysis (nongeographic osteolysis); and C: radiographically occult (Fig. 7) [53]. This modified system shows better correlation of tumor grade with biologic activity and risk of malignancy (grade I usually benign, grade II moderate risk of malignancy, and grade III high risk of malignancy) [53].

### Cortical involvement and extra-osseous component

Endosteal scalloping is erosion of the inner surface of the cortex due to a slow-growing intramedullary process [19]. If the bone has time to produce new periosteum as the inner surface is being eroded, this will result in cortical expansion (also known as insufflation or ballooning) [19]. These processes are typically seen with slow-growing benign tumors that allow for remodeling of the cortex as the tumor expands, such as fibroxanthoma, fibrous dysplasia, unicameral or aneurysmal bone cysts, giant cell tumor, and chondromyxoid fibroma [14, 19]. However, notable malignant exceptions include low grade chondrosarcoma and some slow-growing metastases, such as renal cell and thyroid carcinoma [14]. With slow-growing non-aggressive lesions, the cortex may have normal thickness or be only mildly thinned. With more aggressive lesions, there can be focal cortical interruptions or eventually more wide-spread cortical destruction. These findings can typically be seen with high-grade malignant lesions or aggressive osteomyelitis, and can allow for growth of an extra-osseous soft tissue component through these defects [19]. Some small round blue cell tumors such as Ewing sarcoma and non-Hodgkin’s lymphoma can have permeative growth through Haversian canals that may lead to sizeable soft-tissue component without radiographic evidence of significant cortical destructive change [54]. Because radiography has a low sensitivity for soft tissue extension, cross-sectional imaging is generally warranted for detection and characterization of a suspected soft tissue component, with MRI the preferred modality [15].

### Periosteal reaction — aggressive vs non-aggressive

**Fig. 8:**
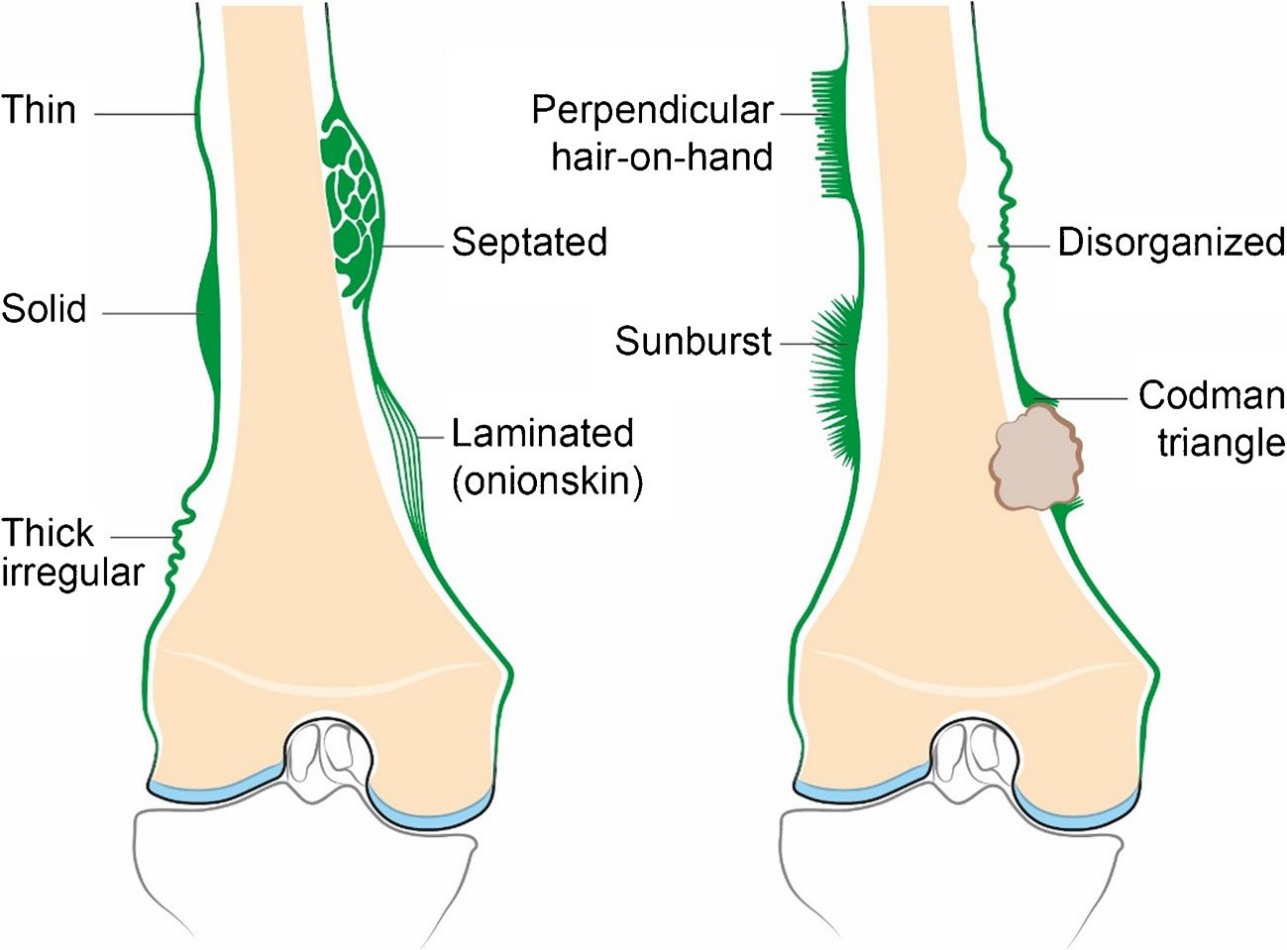
Illustration demonstrating non-aggressive (left) versus aggressive (right) patterns of periosteal reaction

Periosteal reaction occurs when cortical bone reacts to an insult, such as from tumor, infection, trauma, certain drugs, and some arthritic conditions. This can manifest as elevation of the periosteum from the cortex, with the appearance determined by the intensity, aggressiveness, and duration of the insult [55]. The radiographic appearance of periosteal reaction is typically divided into two main types: aggressive versus non-aggressive (Fig. 8).

Non-aggressive periosteal reaction indicates a less intense and slowly progressive process and may have a thin, solid, thick irregular, septated, or shell appearance [55]. Thin periosteal reaction is produced by conditions that induce continuous hyperemia and modulate inactive fibroblasts of the outer layer into osteoblastic cells resulting in a thin (1–2 mm) layer of new bone adjacent to the cortex [56]. Thin periosteal reaction can be physiologic in premature infants up to 6 months of age and can additionally be seen with early fracture healing, osteomyelitis, and malignant lesions (including Ewing sarcoma and osteosarcoma) [56]. Solid periosteal new bone formation occurs when a slow-growing benign process allows for multiple lamellations of periosteal reaction to coalesce and ossify into a continuous solid layer, typically secondary to benign tumors such as osteoid osteoma or with osteomyelitis; malignant causes are rare [56]. Thick irregular periostitis may imply a temporally and spatially heterogenous process, such as chronic osteomyelitis or chronic venous stasis [57, 58]. Septated periosteal reaction is similar in appearance to solid along the outer surface, though also features non-ossified pockets in the reactive bone beneath the surface. The shell pattern occurs when a slowly growing central lytic lesion is surrounded by a rim of periosteal reaction, as can be seen with giant cell tumor and aneurysmal bone cyst, with the thickness of the shell inversely proportional to the rate of lesion expansion [57].

**Fig. 9:**
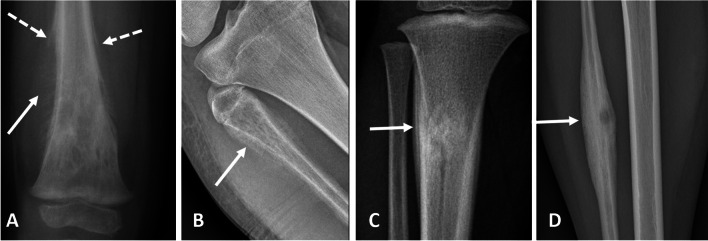
Different types of periosteal reactions. **a** Codman triangle (dotted arrows) plus sunburst (arrows), aggressive, final diagnosis Ewing sarcoma). **b** Disorganized (arrow — aggressive, final diagnosis Ewing sarcoma). **c** Multilayered “onion-skin” (arrow — aggressive, final diagnosis osteosarcoma). **d** Solid (arrow — non-aggressive, final diagnosis osteoid osteoma)

Aggressive periosteal reaction patterns include laminated (onion skin), spiculated, disorganized, and Codman triangle [55]. With laminated periostitis, alternating cycles of rapid and slow injury to bone (intermittent or discontinuous growth) result in the formation of concentric layers (“onion skin” appearance), as can be seen with sarcomas, osteomyelitis, and chondroblastomas [55, 57]. Spiculated periostitis results from rapid and continuous processes that produce spicules of periosteal new bone formation perpendicular to the cortex (“hair-on-end” pattern, typical of Ewing sarcoma) or radiating from a central source (divergent or “sunburst” pattern). These can be populated with malignant cells, and in the case of osteosarcoma, osteoid matrix formation can increase the density of the periosteal reaction [55, 57]. A Codman triangle is a type of interrupted periosteal reaction that results when rapidly growing process only allow the peripheral margin time to form periosteal reaction, which then arises from the cortex at an angle [15, 59]. A Codman triangle is classically seen with osteosarcoma but can also be seen occasionally with infection and metastases [55]. In Fig. 9, different types of aggressive and non-aggressive periosteal reactions are shown.

Multiple periosteal reaction pattern types can be seen simultaneously associated with a single lesion, with the presence of both aggressive and non-aggressive types indicating heterogeneity of the underlying process. The overall aggressivity should be based on the most aggressive type [59]. Multifocal or diffuse periosteal reaction has an extensive differential diagnosis that includes traumatic, neoplastic, congenital, inflammatory, vascular, metabolic, and drug-related causes, with neoplastic causes including metastatic disease, Langerhans cell histiocytosis, leukemia, and lymphoma [57].

### Tumor matrix and mineralization

**Fig. 10:**
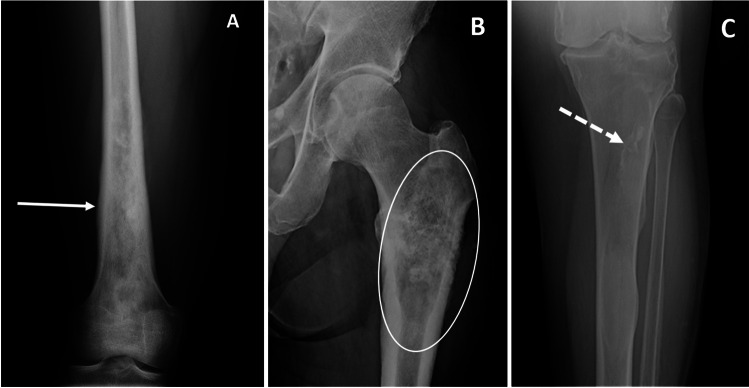
Radiographic examples of osteoid (**a** arrow — osteosarcoma distal femur), chondroid (**b** circled area — chondrosarcoma proximal femur), and fibrous (**c** dotted arrow — fibrous dysplasia proximal tibia) matrix

Tumor matrix represents the type of acellular material produced by tumor cells, which can be categorized as osteoid, chondroid, or fibrous matrix (Fig. 10) [26]. Tumors can also demonstrate cystic, necrotic, and hemorrhagic components, as well as adipocytic healing. The radiographic appearance of the tumor matrix can be helpful for formulating a differential diagnosis [19]. Osteoid matrix may have a fluffy, amorphous, cloud-like, or dense mineralization and is characteristic of bone-forming tumors such as osteoid osteoma, osteoblastoma, and osteosarcoma [19]. Chondroid matrix can have a flocculent, comma-shaped, popcorn, or ring-and-arc appearance and can occur with cartilaginous tumors such as enchondroma, chondroblastoma, and chondrosarcoma [19]. Fibrous matrix can have a frosty or hazy (ground-glass) appearance and is characteristic of fibrous dysplasia, although is occasionally seen in other fibrous tumors such as ossifying fibroma. However, many lesions do not demonstrate matrix mineralization, including some osteoid, chondroid, or fibrous tumors; therefore, the absence of a characteristic matrix mineralization does not exclude these tumors [15].

### Pathological fracture and impending fracture (Mirels’ criteria)

**Fig. 11:**
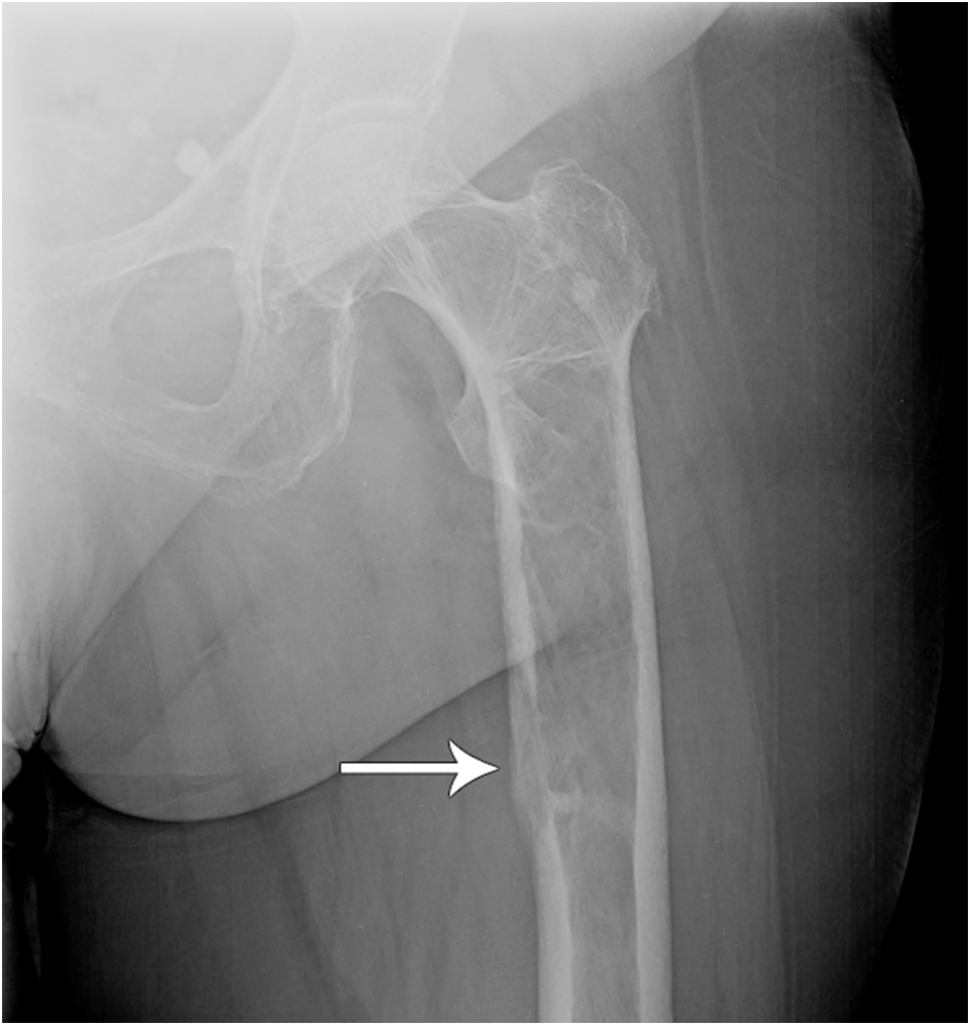
AP radiograph of the left hip demonstrates a hemangioma that is lytic, peritrochanteric, and involves greater than 2/3 of the bone width, including medial cortical involvement (*arrow*). Even without information regarding pain, this lesion would have a minimum Mirels’ score of 9 and therefore should undergo prophylactic fixation

Radiographic evaluation of bone lesions is also important to assess for impending fracture risk, as prospective identification and prophylactic fixation may reduce morbidity. Harrington initially proposed criteria that indicate a high risk for pathological fracture to include: greater than 50% cortical destruction, persistent pain with weight-bearing or after radiotherapy, and proximal femur lesions greater than 2.5 cm or associated with lesser trochanter avulsion [60]. In 1989, Mirels proposed a scoring system to better classify impending pathological fracture risk based on the site, density, and size (width of involvement) of the lesion and degree of pain, each on a 1–3 scale for a total score of 3–12 (Fig. 11, Table 1) [61]. Based on an analysis of 78 bone lesions, it was proposed that a score of 7 or less is low risk for impending fracture and may be treated conservatively; a score of 9 or higher is at high risk for impending fracture and should undergo prophylactic fixation; and a score of 8 should be judged clinically weighing the risks of prophylactic surgery versus impending fracture [61]. In a study of 92 patients with femoral lesions that underwent prophylactic stabilization, 85% were lytic, 66% were peritrochanteric, 73% reported functional pain, and 90% had a Mirels score greater than 8 [62].

**Table 1:** Mirels’ Scoring System

Variable	Score		
	1	2	3
Site	Upper limb	Lower limb	Peritrochanteric
Density	Blastic	Mixed	Lytic
Size (bone width)	< 1/3	1/3–2/3	> 2/3
Pain	Mild	Moderate	Functional

## Sclerotic bone lesions

**Fig. 12:**
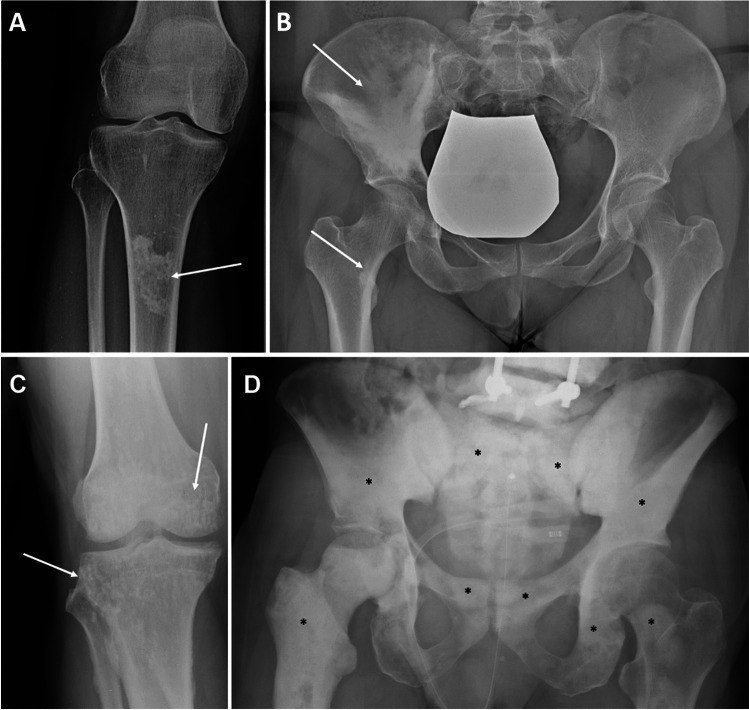
Examples of sclerotic bone lesions: bone infarct (**a**, *arrow*), melorheostosis (**b**, *arrows*), osteopoikilosis (**c**, *arrows*), prostate cancer metastases (**d**, *asterisks*)

A separate analysis should be performed for sclerotic bone lesions. Sclerosis is a region of bone that shows up as denser than the surrounding trabecular bone on radiography or CT scans. The Lodwick grading method is not applied when assessing focal sclerotic lesions. Nonetheless, the initial assessment of a sclerotic lesion should start with a look for concerning features, such as the presence of pain or aggressive behavior on imaging (e.g., pathologic fracture, soft tissue extension, cortical involvement, or aggressive periosteal reaction). This is similar to the approach for a lucent lesion. When one or more of these characteristics are present, there may be cause for concern, such as infection, primary bone malignant tumors, or metastases [63]. Examples of various sclerotic lesions are depicted in Fig. 12.

In the event that the lesion exhibits no alarming characteristics, but the patient has a history of cancer, further testing (MRI or 18F-FDG PET/CT, for example) or imaging surveillance may be necessary. A few primary tumors, such as those of the prostate, breast, lung, gastrointestinal tract, carcinoid, and transitional cell carcinoma, can result in only sclerotic bone metastases. Prostate and breast cancer are the most prevalent types [64]. Features that are very indicative of benign entities, such as non-ossifying fibroma (sclerotic in adult age) or osteoma, may be comforting in lesions without worrisome symptoms and in individuals without a known cancer prone to metastasis to the bone [63].

With a reported frequency of 14% on autopsy series (but up to 89% prevalence on CTs for polytrauma), enostosis, also known as bone island (BI), is the most frequent incidental sclerotic bone lesion [64, 65] BI can occur anywhere in the skeleton with plain radiographs showing uniformly sclerosis with characteristic bony streaks radiating outward (“thorny radiation”) that blend with the trabeculae and create a feathered or brush-like border. Most BI range in size from 1 mm to 2 cm, although “giant” bone islands larger than 2 cm have also been reported [66]. When followed-up on serial imaging, up to 31% of BI will change in size; however, their growth is expected to be less than 25% in diameter over 6 months or less than 50% in 1 year. Biopsy should be considered for lesions exceeding this growth rate [64].

Non-ossifying fibroma (NOF) affects 30% of children but is frequently undiagnosed [67]. NOF is often located at the metaphysis, eccentrically in the subcortical bone marrow and typically exhibits a lucent or mixed pattern in childhood, but commonly ossifies and become homogeneously sclerotic after puberty, and eventually disappears (stage D) [68].

Osteomas are another benign sclerotic bone lesion nearly exclusively seen in the craniofacial region. They frequently affect the jaws, maxilla, and paranasal sinuses with a very slow but continuous growth pattern [69]. Radiographically, osteomas appear as dense lesions similar to bone cortex and may cause bone expansion [69].

Osteoid osteoma (OO) accounts for 10% of primary bone tumors and is the third most frequent benign bone tumor, often presenting as a cortically based sclerotic lesion [70]. Three concentric components make up an OO: a fibrovascular rim, a radiolucent nidus that represents the neoplastic activity, and surrounding reactive sclerosis; however, on x-rays, dense sclerosis may hide the nidus, presenting as regular solid periosteal reaction or focal cortical thickening [71].

Bone infarcts (late phase), stress fractures, and chronic osteomyelitis are non-tumoral diseases that can also manifest as focal sclerotic bone regions. Older (late phase) bone infarcts typically emerge as sclerotic, inhomogeneous, serpiginous lesions near the metaphysis of the long bones [71]. Bone sclerosis is frequently linked to periosteal response and cortical thickening in stress fractures and chronic osteomyelitis [71].

In a patient with multiple sclerotic bone lesions, the possibility of metastatic bone disease should be excluded, considering the characteristics of the lesions and the age, sex, and clinical history. In older male patients, a prostate specific antigen (PSA) test, and in female patients, mammography should be reviewed. Bone scintigraphy should be considered to help differentiate bone islands from sclerotic metastases or for patients with a history of malignancies that are likely to have sclerotic metastases (e.g., prostate, breast, and uroepithelial tumors), with a sensitivity for an individual sclerotic prostate metastasis of 59%, which improves to 90% with SPECT imaging [72, 73].

Several non-malignant conditions can also produce multiple sclerotic bone lesions. Osteopoikilosis is defined by the presence of several enostoses, which are usually found around joints and oriented parallel to the surrounding trabeculae [45]. Tuberous sclerosis complex (TSC) has multiple sclerotic bone lesions in 89% of patients [74]. POEMS syndrome (Polyneuropathy, Organomegaly, Endocrinopathy, Monoclonal plasma cell disorder, Skin alterations) is frequently associated with sclerotic bone lesions, which are typically smaller than 1 cm in diameter [75]. Melorheostosis can present with multiple sclerotic lesion, typically on one side of the cortical bone, giving the impression of “dripping candle wax” on radiographs [45]. The blastic phase of Paget disease presents with osseous sclerosis, trabecular coarsening, cortical thickening, and bone expansion [76].

## Future perspectives

Although conventional radiography remains the current gold standard for the initial imaging evaluation of bone lesions and likely will continue to play a critical role in the foreseeable future, imaging and technical advances will also have an increasingly important role in bone lesion evaluation. Artificial intelligence is being applied to conventional radiography for automated bone lesion detection and classification (e.g., differentiation between benign and malignant) [77, 78]. In a meta-analysis, the pooled sensitivity and specificity for AI algorithms for primary bone tumor detection were 84% and 91%, respectively, which is nearly as good as those for clinicians, which were 85% and 94%, respectively [77]. A systematic review of machine learning algorithms for differentiating between benign and malignant bone lesions overall reported accuracy, sensitivity, and specificity ranges from 0.44 to 0.99, 0.63 to 1.00, and 0.73 to 0.96, respectively, with AUCs of 0.73–0.96 [78]. A deep learning classification of primary bone tumors on radiographs demonstrated model area under curve (AUC) of 0.89 and 0.88 for benign versus non benign on cross-validation and external testing, respectively, with accuracy similar to subspecialists and better than junior radiologists [79]. A recent study used a You Only Look Once (YOLO) deep learning model to both detect and classify (normal, benign, intermediate, or malignant) primary bone tumors on radiographs, with detection accuracies of 86% and 85% on internal and external validation and Cohen kappa scores for classification relative to ground truth of 0.86 and 0.82 at internal and external validation [80].

## Conclusions

Conventional radiographic analysis of bone lesions remains key for initial assessment and approach to subsequent imaging and management. Combining clinical features such as patient age and lesion location with radiographic features such as density (lucent, mixed, or sclerotic) and tumor aggressiveness based on margins and periosteal reaction evaluation, along with tumor matrix and lesion multiplicity, can help determine if it is a benign “do not touch” lesion or a lesion that requires advanced imaging for further evaluation and/or image-guided biopsy for definitive diagnosis.
